# (*E*)-1-(1-Benzyl-5-methyl-1*H*-1,2,3-triazol-4-yl)-3-(4-fluoro­phen­yl)prop-2-en-1-one

**DOI:** 10.1107/S1600536811038943

**Published:** 2011-09-30

**Authors:** Hoong-Kun Fun, Madhukar Hemamalini, Poovan Shanmugavelan, Alagusundaram Ponnuswamy, Rathinavel Jagatheesan

**Affiliations:** a X-ray Crystallography Unit, School of Physics, Universiti Sains Malaysia, 11800 USM, Penang, Malaysia; b Department of Organic Chemistry, School of Chemistry, Madurai Kamaraj University, Madurai 625 021, Tamil Nadu, India; c Department of Chemistry, Thanthai Hans Roever College, Perambalur 621 212, Tamil Nadu, India

## Abstract

The asymmetric unit of the title compound, C_19_H_16_FN_3_O, contains two crystallographically independent mol­ecules. The triazole rings in both mol­ecules are essentially planar with maximum deviations of 0.002 (1) and 0.001 (1) Å. The dihedral angles between the benzene and fluorophenyl rings are 79.36 (9) and 89.40 (10)° in the two molecules. In the crystal, the two independent mol­ecules are linked by C—H⋯N hydrogen bonds, forming dimers. Furthermore, the crystal structure is stabilized by C—H⋯π inter­actions.

## Related literature

For applications of 1,2,3-triazole, see: Banerjee *et al.* (1966 ▶); Laliberte *et al.* (1967 ▶); Suwa *et al.* (1984 ▶). For applications of chalcones, see: Ballesteros *et al.* (1995 ▶); Kothari *et al.* (1999 ▶); Nagaraj & Reddy (2007 ▶). The crystal structure is isomorphous with that of (*E*)-1-(1-benzyl-5-methyl-1*H*-1,2,3-triazol-4-yl)-3-phenyl­prop-2-en-1-one, see: Fun *et al.* (2011 ▶).
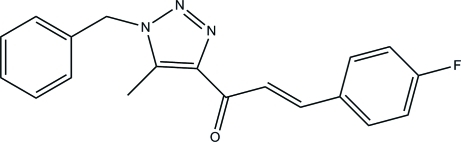


## Experimental

### 

#### Crystal data

C_19_H_16_FN_3_O
*M* *_r_* = 321.35
Monoclinic, 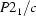

*a* = 12.458 (1) Å
*b* = 13.7528 (11) Å
*c* = 19.3128 (15) Å
β = 100.183 (2)°
*V* = 3256.8 (4) Å^3^
*Z* = 8
Mo *K*α radiation
μ = 0.09 mm^−1^
*T* = 296 K
0.26 × 0.22 × 0.16 mm

#### Data collection

Bruker APEXII DUO CCD area-detector diffractometer
Absorption correction: multi-scan (*SADABS*; Bruker, 2009 ▶) *T*_min_ = 0.977, *T*_max_ = 0.986
37230 measured reflections
9500 independent reflections
4782 reflections with *I* > 2σ(*I*)
*R*_int_ = 0.048

#### Refinement

*R*[*F* ^2^ > 2σ(*F* ^2^)] = 0.049
*wR*(*F* ^2^) = 0.158
*S* = 0.99
9500 reflections
435 parameters
H-atom parameters constrained
Δρ_max_ = 0.16 e Å^−3^
Δρ_min_ = −0.19 e Å^−3^

### 

Data collection: *APEX2* (Bruker, 2009 ▶); cell refinement: *SAINT* (Bruker, 2009 ▶); data reduction: *SAINT*; program(s) used to solve structure: *SHELXTL* (Sheldrick, 2008 ▶); program(s) used to refine structure: *SHELXTL*; molecular graphics: *SHELXTL*; software used to prepare material for publication: *SHELXTL* and *PLATON* (Spek, 2009 ▶).

## Supplementary Material

Crystal structure: contains datablock(s) global, I. DOI: 10.1107/S1600536811038943/rz2639sup1.cif

Structure factors: contains datablock(s) I. DOI: 10.1107/S1600536811038943/rz2639Isup2.hkl

Supplementary material file. DOI: 10.1107/S1600536811038943/rz2639Isup3.cml

Additional supplementary materials:  crystallographic information; 3D view; checkCIF report

## Figures and Tables

**Table 1:** Hydrogen-bond geometry (Å, °) *Cg*3 and *Cg*6 are the centroids of the of the C13*A*–C18*A* and C13*B*–C18*B* rings, respectively.

*D*—H⋯*A*	*D*—H	H⋯*A*	*D*⋯*A*	*D*—H⋯*A*
C12*B*—H12*C*⋯N1*A*^i^	0.97	2.46	3.422 (2)	172
C5*A*—H5*AA*⋯*Cg*6^ii^	0.93	2.91	3.842 (2)	178
C12*A*—H12*A*⋯*Cg*3^iii^	0.97	2.62	3.551 (2)	161

Symmetry codes: (i) 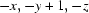; (ii) 
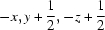
; (iii) 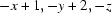.

**Fig. 1.**
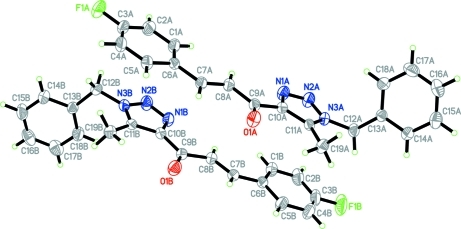
An ORTEP view of the title compound, showing 50% probability displacement ellipsoids.

**Fig. 2.**
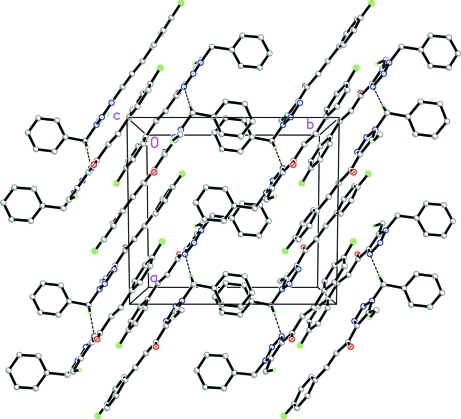
The crystal packing of the title compound. H atoms not involved in hydrogen bonding are omitted.

## supplementary crystallographic information

### Comment

Organic compounds having the 1,2,3-triazole nucleus have the potential to induce antiviral, agonistic, antibacterial, antimicrobial, anti-HIV, anticonvulsant and anti-allergic activities. In addition, compounds having 1,2,3-triazole group have found industrial application such as dyes, corrosion inhibitors, sensors and photo-stabilizers (Banerjee *et al.*, 1966; Laliberte *et al.*, 1967; Suwa *et al.*, 1984). The chalcone skeleton is a unique template for synthesizing various heterocyclic compounds. The compounds with the backbone of chalcones are associated with different biological activities like cardiovascular, antispasmodic, anthelmintics, antiulcer, anti-inflammatory, antiviral, antiallergic, fungicidal, bactericidal, insecticidal, antitumor, herbicidal, anticancer, antitubercular and anti-HIV (Ballesteros *et al.*, 1995; Kothari *et al.*, 1999; Nagaraj & Reddy, 2007).  Chalcones, considered as the precursors of flavonoids and isoflavonoids, are abundant in edible plants, and have also been shown to display a diverse array of pharmacological activities. The presence of a reactive α, β-unsaturated keto function in chalcones is found to be responsible for their activities.

The asymmetric unit of the title compound, contains two crystallographically independent (*E*)-1-(1-benzyl-5-methyl-1*H*-1,2,3-triazol-4-yl)-3-(4-fluorophenyl)prop-2-en-1-one  molecules (A & B) as shown in Fig. 1. In both molecules, the triazole (N1A–N3A/C10A,C11A and N1B–N3B/C10B,C11B) units are essentially planar, with maximum deviations of  0.002 (1) Å for atom C11A and 0.001 (1) Å for atom N3B. The dihedral angles between the phenyl ring and the flurophenyl ring in the molecules A and B are 79.36 (9)° and 89.40 (10)° respectively.

In the crystal, (Fig. 2), the two independent molecules are connected *via* intermolecular C—H···N hydrogen bonds, (Table 1), to form dimers. Furthermore, the crystal structure is stabilized by C—H···π interactions involving the centroids of the C13A–C18A (Cg3) and C13B–C18B (Cg6) rings. This crystal structure is isomorphous with that of (*E*)-1-(1-benzyl-5-methyl-1*H*-1,2,3-triazol-4-yl)-3-phenylprop-2-en-1-one (Fun *et al.*, 2011).

### Experimental

A mixture of 4-acetyl-1-benzyl-5-methyl-1,2,3-triazole (0.20 g, 0.01 mol) and *p*-fluorobenzaldehyde (0.01 mol) was stirred in ethanol (2–3 ml) and then a 50% sodium hydroxide solution (0.5 ml) was added. The mixture was stirred for 2 minutes at room temperature and poured onto excess of crushed ice and neutralized with dilute hydrochloric acid. (*E*)-1-(1- Benzyl-5-methyl-1*H*-1,2,3-triazol-4-yl)-3-(4-fluorophenyl)prop-2-en-1-one, which precipitated out as a solid, was filtered and recrystallized from ethanol. Yield: 0.29 g (98%), Mp.169–170°C.

### Refinement

All hydrogen atoms were positioned geometrically [C–H = 0.93–0.97 Å] and were refined using a riding model, with *U*_iso_(H) = 1.2 or 1.5 *U*_eq_(C).  A rotating group model was applied to the methyl groups.

### Crystal data

**Table:**

C_19_H_16_FN_3_O	*F*(000) = 1344
*M**_r_* = 321.35	*D*_x_ = 1.311 Mg m^−^^3^
Monoclinic, *P*2_1_/*c*	Mo *K*α radiation, λ = 0.71073 Å
Hall symbol: -P 2ybc	Cell parameters from 5012 reflections
*a* = 12.458 (1) Å	θ = 2.5–28.8°
*b* = 13.7528 (11) Å	µ = 0.09 mm^−^^1^
*c* = 19.3128 (15) Å	*T* = 296 K
β = 100.183 (2)°	Block, colourless
*V* = 3256.8 (4) Å^3^	0.26 × 0.22 × 0.16 mm
*Z* = 8	

### Data collection

**Table:**

Bruker APEXII DUO CCD area-detector diffractometer	9500 independent reflections
Radiation source: fine-focus sealed tube	4782 reflections with *I* > 2σ(*I*)
graphite	*R*_int_ = 0.048
φ and ω scans	θ_max_ = 30.1°, θ_min_ = 1.8°
Absorption correction: multi-scan (*SADABS*; Bruker, 2009)	*h* = −11→17
*T*_min_ = 0.977, *T*_max_ = 0.986	*k* = −16→19
37230 measured reflections	*l* = −27→27

### Refinement

**Table:**

Refinement on *F*^2^	Primary atom site location: structure-invariant direct methods
Least-squares matrix: full	Secondary atom site location: difference Fourier map
*R*[*F*^2^ > 2σ(*F*^2^)] = 0.049	Hydrogen site location: inferred from neighbouring sites
*wR*(*F*^2^) = 0.158	H-atom parameters constrained
*S* = 0.99	*w* = 1/[σ^2^(*F*_o_^2^) + (0.0702*P*)^2^ + 0.2551*P*] where *P* = (*F*_o_^2^ + 2*F*_c_^2^)/3
9500 reflections	(Δ/σ)_max_ = 0.001
435 parameters	Δρ_max_ = 0.16 e Å^−^^3^
0 restraints	Δρ_min_ = −0.19 e Å^−^^3^

### Special details

**Table:**

Geometry. All s.u.'s (except the s.u. in the dihedral angle between two l.s. planes) are estimated using the full covariance matrix. The cell s.u.'s are taken into account individually in the estimation of s.u.'s in distances, angles and torsion angles; correlations between s.u.'s in cell parameters are only used when they are defined by crystal symmetry. An approximate (isotropic) treatment of cell s.u.'s is used for estimating s.u.'s involving l.s. planes.
Refinement. Refinement of F^2^ against ALL reflections. The weighted R-factor wR and goodness of fit S are based on F^2^, conventional R-factors R are based on F, with F set to zero for negative F^2^. The threshold expression of F^2^ > 2σ(F^2^) is used only for calculating R-factors(gt) etc. and is not relevant to the choice of reflections for refinement. R-factors based on F^2^ are statistically about twice as large as those based on F, and R- factors based on ALL data will be even larger.

### Fractional atomic coordinates and isotropic or equivalent isotropic displacement parameters (Å^2^)

**Table:**

	*x*	*y*	*z*	*U*_iso_*/*U*_eq_	
F1A	−0.32640 (10)	0.37115 (10)	0.09739 (9)	0.1059 (5)	
O1A	0.23331 (11)	0.69816 (10)	0.24388 (6)	0.0639 (4)	
N1A	0.24431 (12)	0.74029 (11)	0.06245 (7)	0.0589 (4)	
N2A	0.31516 (13)	0.79113 (12)	0.03501 (8)	0.0630 (4)	
N3A	0.38780 (11)	0.82740 (9)	0.08961 (7)	0.0468 (3)	
C1A	−0.08988 (15)	0.51783 (14)	0.08771 (10)	0.0588 (5)	
H1AA	−0.0572	0.5484	0.0538	0.071*	
C2A	−0.18483 (17)	0.46491 (15)	0.06714 (12)	0.0710 (6)	
H2AA	−0.2156	0.4589	0.0198	0.085*	
C3A	−0.23210 (15)	0.42178 (13)	0.11782 (13)	0.0669 (5)	
C4A	−0.18837 (16)	0.42645 (14)	0.18714 (12)	0.0693 (6)	
H4AA	−0.2213	0.3948	0.2204	0.083*	
C5A	−0.09391 (15)	0.47917 (13)	0.20721 (10)	0.0576 (4)	
H5AA	−0.0637	0.4836	0.2547	0.069*	
C6A	−0.04287 (13)	0.52584 (11)	0.15815 (9)	0.0443 (4)	
C7A	0.05598 (13)	0.58185 (11)	0.18279 (9)	0.0458 (4)	
H7AA	0.0832	0.5787	0.2308	0.055*	
C8A	0.11174 (13)	0.63664 (12)	0.14531 (9)	0.0474 (4)	
H8AA	0.0903	0.6399	0.0967	0.057*	
C9A	0.20747 (13)	0.69246 (11)	0.17970 (9)	0.0456 (4)	
C10A	0.27110 (13)	0.74407 (11)	0.13399 (8)	0.0427 (4)	
C11A	0.36359 (12)	0.80044 (10)	0.15187 (8)	0.0408 (3)	
C12A	0.47819 (14)	0.88739 (12)	0.07504 (10)	0.0529 (4)	
H12A	0.4864	0.8776	0.0265	0.063*	
H12B	0.5450	0.8659	0.1048	0.063*	
C13A	0.46318 (13)	0.99394 (12)	0.08708 (8)	0.0449 (4)	
C14A	0.55168 (15)	1.04882 (13)	0.11866 (9)	0.0538 (4)	
H14A	0.6180	1.0183	0.1348	0.065*	
C15A	0.54284 (19)	1.14811 (15)	0.12649 (10)	0.0689 (5)	
H15A	0.6034	1.1843	0.1466	0.083*	
C16A	0.4451 (2)	1.19308 (15)	0.10462 (11)	0.0729 (6)	
H16A	0.4386	1.2596	0.1114	0.087*	
C17A	0.35653 (19)	1.14075 (16)	0.07276 (12)	0.0755 (6)	
H17A	0.2905	1.1720	0.0573	0.091*	
C18A	0.36500 (15)	1.04084 (14)	0.06344 (10)	0.0618 (5)	
H18A	0.3049	1.0056	0.0414	0.074*	
C19A	0.42936 (15)	0.83060 (14)	0.22006 (9)	0.0578 (5)	
H19A	0.5055	0.8245	0.2180	0.087*	
H19B	0.4120	0.7898	0.2569	0.087*	
H19C	0.4132	0.8970	0.2295	0.087*	
F1B	0.73003 (13)	0.72639 (11)	0.08097 (11)	0.1253 (6)	
O1B	0.27643 (11)	0.39555 (10)	0.25084 (6)	0.0671 (4)	
N1B	0.15078 (13)	0.35495 (11)	0.07189 (8)	0.0592 (4)	
N2B	0.06363 (14)	0.30509 (12)	0.04708 (8)	0.0655 (4)	
N3B	0.02495 (11)	0.26840 (10)	0.10339 (7)	0.0519 (4)	
C1OB	0.16882 (13)	0.35062 (11)	0.14372 (8)	0.0457 (4)	
C1B	0.49013 (17)	0.58159 (14)	0.08252 (11)	0.0667 (5)	
H1BA	0.4331	0.5547	0.0507	0.080*	
C2B	0.5694 (2)	0.63425 (16)	0.05776 (13)	0.0829 (7)	
H2BA	0.5667	0.6428	0.0097	0.099*	
C3B	0.65206 (19)	0.67376 (15)	0.10544 (16)	0.0807 (7)	
C4B	0.65816 (18)	0.66406 (16)	0.17544 (16)	0.0833 (7)	
H4BA	0.7146	0.6928	0.2067	0.100*	
C5B	0.57889 (15)	0.61067 (14)	0.19975 (12)	0.0674 (5)	
H5BA	0.5825	0.6031	0.2480	0.081*	
C6B	0.49368 (13)	0.56785 (11)	0.15370 (9)	0.0493 (4)	
C7B	0.41315 (13)	0.51127 (12)	0.18278 (9)	0.0496 (4)	
H7BA	0.4192	0.5130	0.2315	0.060*	
C8B	0.33261 (14)	0.45779 (12)	0.14874 (9)	0.0511 (4)	
H8BA	0.3211	0.4558	0.0998	0.061*	
C9B	0.26054 (13)	0.40125 (12)	0.18655 (9)	0.0475 (4)	
C11B	0.08781 (13)	0.29476 (11)	0.16430 (8)	0.0445 (4)	
C12B	−0.07283 (14)	0.20804 (13)	0.09197 (11)	0.0602 (5)	
H12C	−0.1147	0.2225	0.0459	0.072*	
H12D	−0.1173	0.2251	0.1266	0.072*	
C13B	−0.04989 (14)	0.10044 (12)	0.09659 (8)	0.0485 (4)	
C14B	−0.13479 (17)	0.03893 (15)	0.10314 (11)	0.0703 (6)	
H14B	−0.2023	0.0648	0.1073	0.084*	
C15B	−0.1208 (2)	−0.06059 (16)	0.10362 (13)	0.0865 (7)	
H15B	−0.1790	−0.1012	0.1081	0.104*	
C16B	−0.0227 (2)	−0.09977 (16)	0.09764 (11)	0.0815 (7)	
H16B	−0.0140	−0.1669	0.0972	0.098*	
C17B	0.0622 (2)	−0.04041 (16)	0.09231 (12)	0.0795 (6)	
H17B	0.1296	−0.0670	0.0888	0.095*	
C18B	0.04912 (17)	0.05965 (15)	0.09199 (11)	0.0672 (5)	
H18B	0.1082	0.0996	0.0886	0.081*	
C19B	0.06490 (15)	0.26518 (14)	0.23379 (10)	0.0615 (5)	
H19D	−0.0115	0.2729	0.2345	0.092*	
H19E	0.1062	0.3051	0.2697	0.092*	
H19F	0.0850	0.1983	0.2423	0.092*	

### Atomic displacement parameters (Å^2^)

**Table:**

	*U*^11^	*U*^22^	*U*^33^	*U*^12^	*U*^13^	*U*^23^
F1A	0.0656 (8)	0.0858 (9)	0.1618 (14)	−0.0349 (7)	0.0079 (8)	−0.0050 (9)
O1A	0.0682 (8)	0.0721 (9)	0.0517 (7)	−0.0202 (7)	0.0112 (6)	−0.0022 (6)
N1A	0.0605 (9)	0.0638 (10)	0.0503 (8)	−0.0176 (8)	0.0043 (7)	−0.0003 (7)
N2A	0.0675 (10)	0.0705 (10)	0.0498 (8)	−0.0217 (8)	0.0067 (7)	0.0012 (7)
N3A	0.0483 (8)	0.0418 (7)	0.0513 (8)	−0.0087 (6)	0.0116 (6)	−0.0008 (6)
C1A	0.0569 (11)	0.0603 (11)	0.0593 (11)	−0.0115 (9)	0.0107 (9)	0.0058 (9)
C2A	0.0655 (13)	0.0687 (13)	0.0746 (13)	−0.0138 (10)	0.0008 (10)	−0.0025 (10)
C3A	0.0474 (10)	0.0463 (10)	0.1058 (17)	−0.0100 (8)	0.0106 (11)	−0.0024 (10)
C4A	0.0607 (12)	0.0569 (12)	0.0966 (16)	−0.0093 (10)	0.0309 (11)	0.0143 (11)
C5A	0.0572 (11)	0.0540 (10)	0.0639 (11)	−0.0057 (9)	0.0171 (9)	0.0092 (8)
C6A	0.0436 (9)	0.0342 (8)	0.0565 (10)	0.0009 (7)	0.0125 (7)	0.0026 (7)
C7A	0.0446 (9)	0.0414 (8)	0.0528 (9)	0.0008 (7)	0.0128 (7)	−0.0001 (7)
C8A	0.0470 (9)	0.0438 (9)	0.0521 (9)	−0.0061 (7)	0.0105 (7)	−0.0017 (7)
C9A	0.0459 (9)	0.0391 (8)	0.0524 (10)	−0.0018 (7)	0.0102 (7)	−0.0015 (7)
C10A	0.0420 (8)	0.0382 (8)	0.0469 (9)	−0.0019 (7)	0.0047 (7)	0.0008 (7)
C11A	0.0419 (8)	0.0337 (7)	0.0471 (8)	−0.0003 (6)	0.0089 (7)	−0.0002 (6)
C12A	0.0509 (10)	0.0477 (10)	0.0643 (11)	−0.0064 (8)	0.0217 (8)	0.0028 (8)
C13A	0.0448 (9)	0.0461 (9)	0.0463 (8)	−0.0051 (7)	0.0146 (7)	0.0049 (7)
C14A	0.0514 (10)	0.0583 (11)	0.0512 (9)	−0.0069 (8)	0.0083 (8)	0.0000 (8)
C15A	0.0820 (15)	0.0608 (12)	0.0643 (12)	−0.0183 (11)	0.0141 (11)	−0.0139 (10)
C16A	0.1014 (18)	0.0482 (11)	0.0758 (13)	−0.0028 (12)	0.0340 (13)	−0.0037 (10)
C17A	0.0717 (14)	0.0663 (14)	0.0938 (16)	0.0204 (11)	0.0288 (12)	0.0190 (11)
C18A	0.0478 (10)	0.0605 (12)	0.0775 (13)	−0.0021 (9)	0.0121 (9)	0.0093 (10)
C19A	0.0532 (10)	0.0644 (11)	0.0545 (10)	−0.0138 (9)	0.0058 (8)	−0.0048 (8)
F1B	0.1008 (11)	0.0844 (10)	0.2067 (18)	−0.0360 (8)	0.0705 (11)	0.0100 (10)
O1B	0.0653 (8)	0.0783 (9)	0.0552 (8)	−0.0194 (7)	0.0037 (6)	0.0041 (6)
N1B	0.0631 (10)	0.0599 (9)	0.0537 (9)	−0.0172 (8)	0.0082 (7)	0.0033 (7)
N2B	0.0758 (11)	0.0642 (10)	0.0533 (9)	−0.0206 (9)	0.0028 (8)	0.0022 (7)
N3B	0.0494 (8)	0.0445 (8)	0.0583 (9)	−0.0085 (6)	0.0002 (7)	0.0061 (6)
C1OB	0.0474 (9)	0.0397 (8)	0.0495 (9)	−0.0019 (7)	0.0072 (7)	0.0037 (7)
C1B	0.0662 (12)	0.0567 (11)	0.0759 (13)	−0.0138 (10)	0.0093 (10)	0.0042 (10)
C2B	0.0942 (17)	0.0646 (13)	0.0970 (17)	−0.0146 (12)	0.0364 (14)	0.0121 (12)
C3B	0.0668 (14)	0.0445 (11)	0.140 (2)	−0.0101 (10)	0.0443 (15)	0.0029 (13)
C4B	0.0537 (12)	0.0662 (14)	0.129 (2)	−0.0188 (10)	0.0142 (13)	−0.0144 (14)
C5B	0.0550 (11)	0.0601 (12)	0.0848 (14)	−0.0092 (9)	0.0065 (10)	−0.0102 (10)
C6B	0.0433 (9)	0.0367 (8)	0.0676 (11)	0.0015 (7)	0.0089 (8)	−0.0003 (7)
C7B	0.0450 (9)	0.0429 (9)	0.0600 (10)	0.0007 (7)	0.0066 (8)	0.0016 (7)
C8B	0.0483 (9)	0.0468 (9)	0.0571 (10)	−0.0054 (7)	0.0067 (8)	0.0021 (8)
C9B	0.0441 (9)	0.0417 (9)	0.0557 (10)	−0.0016 (7)	0.0060 (7)	0.0022 (7)
C11B	0.0417 (8)	0.0365 (8)	0.0535 (9)	0.0010 (7)	0.0037 (7)	0.0045 (7)
C12B	0.0439 (10)	0.0523 (10)	0.0793 (13)	−0.0100 (8)	−0.0035 (9)	0.0028 (9)
C13B	0.0480 (9)	0.0485 (9)	0.0474 (9)	−0.0076 (8)	0.0044 (7)	−0.0003 (7)
C14B	0.0580 (12)	0.0629 (13)	0.0883 (14)	−0.0164 (10)	0.0080 (10)	0.0067 (11)
C15B	0.0947 (18)	0.0572 (13)	0.1027 (18)	−0.0305 (13)	0.0042 (14)	0.0116 (12)
C16B	0.128 (2)	0.0461 (11)	0.0689 (13)	−0.0041 (14)	0.0131 (13)	−0.0026 (10)
C17B	0.1003 (18)	0.0624 (14)	0.0829 (15)	0.0143 (13)	0.0352 (13)	−0.0006 (11)
C18B	0.0647 (12)	0.0610 (12)	0.0810 (13)	−0.0063 (10)	0.0270 (10)	0.0033 (10)
C19B	0.0524 (11)	0.0676 (12)	0.0647 (11)	−0.0037 (9)	0.0113 (9)	0.0163 (9)

### Geometric parameters (Å, °)

**Table:**

F1A—C3A	1.363 (2)	F1B—C3B	1.361 (2)
O1A—C9A	1.2267 (19)	O1B—C9B	1.2248 (19)
N1A—N2A	1.309 (2)	N1B—N2B	1.302 (2)
N1A—C10A	1.364 (2)	N1B—C1OB	1.367 (2)
N2A—N3A	1.3573 (19)	N2B—N3B	1.362 (2)
N3A—C11A	1.343 (2)	N3B—C11B	1.342 (2)
N3A—C12A	1.463 (2)	N3B—C12B	1.458 (2)
C1A—C2A	1.386 (3)	C1OB—C11B	1.381 (2)
C1A—C6A	1.387 (2)	C1OB—C9B	1.463 (2)
C1A—H1AA	0.9300	C1B—C2B	1.376 (3)
C2A—C3A	1.364 (3)	C1B—C6B	1.381 (3)
C2A—H2AA	0.9300	C1B—H1BA	0.9300
C3A—C4A	1.355 (3)	C2B—C3B	1.367 (3)
C4A—C5A	1.378 (3)	C2B—H2BA	0.9300
C4A—H4AA	0.9300	C3B—C4B	1.347 (3)
C5A—C6A	1.388 (2)	C4B—C5B	1.378 (3)
C5A—H5AA	0.9300	C4B—H4BA	0.9300
C6A—C7A	1.459 (2)	C5B—C6B	1.389 (2)
C7A—C8A	1.324 (2)	C5B—H5BA	0.9300
C7A—H7AA	0.9300	C6B—C7B	1.458 (2)
C8A—C9A	1.474 (2)	C7B—C8B	1.322 (2)
C8A—H8AA	0.9300	C7B—H7BA	0.9300
C9A—C10A	1.469 (2)	C8B—C9B	1.475 (2)
C10A—C11A	1.381 (2)	C8B—H8BA	0.9300
C11A—C19A	1.482 (2)	C11B—C19B	1.478 (2)
C12A—C13A	1.501 (2)	C12B—C13B	1.507 (2)
C12A—H12A	0.9700	C12B—H12C	0.9700
C12A—H12B	0.9700	C12B—H12D	0.9700
C13A—C14A	1.385 (2)	C13B—C18B	1.372 (3)
C13A—C18A	1.387 (2)	C13B—C14B	1.378 (2)
C14A—C15A	1.380 (3)	C14B—C15B	1.379 (3)
C14A—H14A	0.9300	C14B—H14B	0.9300
C15A—C16A	1.365 (3)	C15B—C16B	1.359 (3)
C15A—H15A	0.9300	C15B—H15B	0.9300
C16A—C17A	1.369 (3)	C16B—C17B	1.354 (3)
C16A—H16A	0.9300	C16B—H16B	0.9300
C17A—C18A	1.392 (3)	C17B—C18B	1.385 (3)
C17A—H17A	0.9300	C17B—H17B	0.9300
C18A—H18A	0.9300	C18B—H18B	0.9300
C19A—H19A	0.9600	C19B—H19D	0.9600
C19A—H19B	0.9600	C19B—H19E	0.9600
C19A—H19C	0.9600	C19B—H19F	0.9600
			
N2A—N1A—C10A	109.14 (14)	N2B—N1B—C1OB	109.11 (14)
N1A—N2A—N3A	106.65 (13)	N1B—N2B—N3B	106.92 (13)
C11A—N3A—N2A	111.71 (13)	C11B—N3B—N2B	111.45 (13)
C11A—N3A—C12A	129.08 (14)	C11B—N3B—C12B	128.94 (15)
N2A—N3A—C12A	119.21 (13)	N2B—N3B—C12B	119.60 (14)
C2A—C1A—C6A	120.92 (17)	N1B—C1OB—C11B	108.59 (14)
C2A—C1A—H1AA	119.5	N1B—C1OB—C9B	121.66 (15)
C6A—C1A—H1AA	119.5	C11B—C1OB—C9B	129.74 (15)
C3A—C2A—C1A	118.53 (19)	C2B—C1B—C6B	121.3 (2)
C3A—C2A—H2AA	120.7	C2B—C1B—H1BA	119.4
C1A—C2A—H2AA	120.7	C6B—C1B—H1BA	119.4
C4A—C3A—F1A	119.1 (2)	C3B—C2B—C1B	118.4 (2)
C4A—C3A—C2A	122.63 (18)	C3B—C2B—H2BA	120.8
F1A—C3A—C2A	118.3 (2)	C1B—C2B—H2BA	120.8
C3A—C4A—C5A	118.48 (18)	C4B—C3B—F1B	118.9 (2)
C3A—C4A—H4AA	120.8	C4B—C3B—C2B	122.6 (2)
C5A—C4A—H4AA	120.8	F1B—C3B—C2B	118.5 (3)
C4A—C5A—C6A	121.52 (18)	C3B—C4B—C5B	118.6 (2)
C4A—C5A—H5AA	119.2	C3B—C4B—H4BA	120.7
C6A—C5A—H5AA	119.2	C5B—C4B—H4BA	120.7
C1A—C6A—C5A	117.89 (16)	C4B—C5B—C6B	121.3 (2)
C1A—C6A—C7A	123.19 (15)	C4B—C5B—H5BA	119.3
C5A—C6A—C7A	118.92 (15)	C6B—C5B—H5BA	119.3
C8A—C7A—C6A	128.06 (16)	C1B—C6B—C5B	117.75 (17)
C8A—C7A—H7AA	116.0	C1B—C6B—C7B	123.60 (16)
C6A—C7A—H7AA	116.0	C5B—C6B—C7B	118.65 (17)
C7A—C8A—C9A	120.73 (15)	C8B—C7B—C6B	128.24 (17)
C7A—C8A—H8AA	119.6	C8B—C7B—H7BA	115.9
C9A—C8A—H8AA	119.6	C6B—C7B—H7BA	115.9
O1A—C9A—C10A	120.21 (14)	C7B—C8B—C9B	121.48 (16)
O1A—C9A—C8A	122.37 (15)	C7B—C8B—H8BA	119.3
C10A—C9A—C8A	117.41 (14)	C9B—C8B—H8BA	119.3
N1A—C10A—C11A	108.58 (14)	O1B—C9B—C1OB	120.85 (15)
N1A—C10A—C9A	121.93 (14)	O1B—C9B—C8B	122.15 (15)
C11A—C10A—C9A	129.48 (14)	C1OB—C9B—C8B	116.99 (15)
N3A—C11A—C10A	103.93 (13)	N3B—C11B—C1OB	103.93 (14)
N3A—C11A—C19A	122.83 (14)	N3B—C11B—C19B	123.02 (15)
C10A—C11A—C19A	133.24 (15)	C1OB—C11B—C19B	133.05 (15)
N3A—C12A—C13A	113.56 (14)	N3B—C12B—C13B	113.92 (14)
N3A—C12A—H12A	108.9	N3B—C12B—H12C	108.8
C13A—C12A—H12A	108.9	C13B—C12B—H12C	108.8
N3A—C12A—H12B	108.9	N3B—C12B—H12D	108.8
C13A—C12A—H12B	108.9	C13B—C12B—H12D	108.8
H12A—C12A—H12B	107.7	H12C—C12B—H12D	107.7
C14A—C13A—C18A	118.58 (16)	C18B—C13B—C14B	117.94 (18)
C14A—C13A—C12A	119.27 (15)	C18B—C13B—C12B	124.12 (16)
C18A—C13A—C12A	122.01 (16)	C14B—C13B—C12B	117.90 (17)
C15A—C14A—C13A	120.96 (18)	C13B—C14B—C15B	120.8 (2)
C15A—C14A—H14A	119.5	C13B—C14B—H14B	119.6
C13A—C14A—H14A	119.5	C15B—C14B—H14B	119.6
C16A—C15A—C14A	119.90 (19)	C16B—C15B—C14B	120.5 (2)
C16A—C15A—H15A	120.0	C16B—C15B—H15B	119.8
C14A—C15A—H15A	120.0	C14B—C15B—H15B	119.8
C15A—C16A—C17A	120.33 (19)	C17B—C16B—C15B	119.6 (2)
C15A—C16A—H16A	119.8	C17B—C16B—H16B	120.2
C17A—C16A—H16A	119.8	C15B—C16B—H16B	120.2
C16A—C17A—C18A	120.2 (2)	C16B—C17B—C18B	120.4 (2)
C16A—C17A—H17A	119.9	C16B—C17B—H17B	119.8
C18A—C17A—H17A	119.9	C18B—C17B—H17B	119.8
C13A—C18A—C17A	119.94 (19)	C13B—C18B—C17B	120.8 (2)
C13A—C18A—H18A	120.0	C13B—C18B—H18B	119.6
C17A—C18A—H18A	120.0	C17B—C18B—H18B	119.6
C11A—C19A—H19A	109.5	C11B—C19B—H19D	109.5
C11A—C19A—H19B	109.5	C11B—C19B—H19E	109.5
H19A—C19A—H19B	109.5	H19D—C19B—H19E	109.5
C11A—C19A—H19C	109.5	C11B—C19B—H19F	109.5
H19A—C19A—H19C	109.5	H19D—C19B—H19F	109.5
H19B—C19A—H19C	109.5	H19E—C19B—H19F	109.5
			
C10A—N1A—N2A—N3A	0.1 (2)	C1OB—N1B—N2B—N3B	0.1 (2)
N1A—N2A—N3A—C11A	−0.28 (19)	N1B—N2B—N3B—C11B	−0.2 (2)
N1A—N2A—N3A—C12A	179.52 (14)	N1B—N2B—N3B—C12B	−179.56 (15)
C6A—C1A—C2A—C3A	0.9 (3)	N2B—N1B—C1OB—C11B	0.0 (2)
C1A—C2A—C3A—C4A	−1.8 (3)	N2B—N1B—C1OB—C9B	−179.05 (15)
C1A—C2A—C3A—F1A	178.96 (18)	C6B—C1B—C2B—C3B	−0.5 (3)
F1A—C3A—C4A—C5A	−178.99 (17)	C1B—C2B—C3B—C4B	−0.8 (4)
C2A—C3A—C4A—C5A	1.8 (3)	C1B—C2B—C3B—F1B	−179.57 (19)
C3A—C4A—C5A—C6A	−0.8 (3)	F1B—C3B—C4B—C5B	180.00 (19)
C2A—C1A—C6A—C5A	0.0 (3)	C2B—C3B—C4B—C5B	1.2 (4)
C2A—C1A—C6A—C7A	−178.99 (17)	C3B—C4B—C5B—C6B	−0.4 (3)
C4A—C5A—C6A—C1A	−0.1 (3)	C2B—C1B—C6B—C5B	1.3 (3)
C4A—C5A—C6A—C7A	179.01 (17)	C2B—C1B—C6B—C7B	−178.81 (18)
C1A—C6A—C7A—C8A	4.2 (3)	C4B—C5B—C6B—C1B	−0.8 (3)
C5A—C6A—C7A—C8A	−174.79 (17)	C4B—C5B—C6B—C7B	179.25 (18)
C6A—C7A—C8A—C9A	176.88 (15)	C1B—C6B—C7B—C8B	6.9 (3)
C7A—C8A—C9A—O1A	−7.1 (3)	C5B—C6B—C7B—C8B	−173.16 (18)
C7A—C8A—C9A—C10A	174.33 (15)	C6B—C7B—C8B—C9B	177.35 (16)
N2A—N1A—C10A—C11A	0.1 (2)	N1B—C1OB—C9B—O1B	−179.00 (16)
N2A—N1A—C10A—C9A	−178.98 (15)	C11B—C1OB—C9B—O1B	2.2 (3)
O1A—C9A—C10A—N1A	−179.34 (16)	N1B—C1OB—C9B—C8B	0.3 (2)
C8A—C9A—C10A—N1A	−0.7 (2)	C11B—C1OB—C9B—C8B	−178.47 (16)
O1A—C9A—C10A—C11A	1.8 (3)	C7B—C8B—C9B—O1B	−5.3 (3)
C8A—C9A—C10A—C11A	−179.51 (15)	C7B—C8B—C9B—C1OB	175.37 (15)
N2A—N3A—C11A—C10A	0.31 (18)	N2B—N3B—C11B—C1OB	0.14 (18)
C12A—N3A—C11A—C10A	−179.47 (15)	C12B—N3B—C11B—C1OB	179.46 (16)
N2A—N3A—C11A—C19A	−179.59 (15)	N2B—N3B—C11B—C19B	179.98 (16)
C12A—N3A—C11A—C19A	0.6 (3)	C12B—N3B—C11B—C19B	−0.7 (3)
N1A—C10A—C11A—N3A	−0.22 (18)	N1B—C1OB—C11B—N3B	−0.06 (18)
C9A—C10A—C11A—N3A	178.72 (16)	C9B—C1OB—C11B—N3B	178.85 (16)
N1A—C10A—C11A—C19A	179.66 (17)	N1B—C1OB—C11B—C19B	−179.88 (18)
C9A—C10A—C11A—C19A	−1.4 (3)	C9B—C1OB—C11B—C19B	−1.0 (3)
C11A—N3A—C12A—C13A	−75.8 (2)	C11B—N3B—C12B—C13B	−80.5 (2)
N2A—N3A—C12A—C13A	104.47 (17)	N2B—N3B—C12B—C13B	98.73 (19)
N3A—C12A—C13A—C14A	139.38 (16)	N3B—C12B—C13B—C18B	−17.6 (3)
N3A—C12A—C13A—C18A	−44.9 (2)	N3B—C12B—C13B—C14B	165.00 (16)
C18A—C13A—C14A—C15A	−0.1 (3)	C18B—C13B—C14B—C15B	−1.2 (3)
C12A—C13A—C14A—C15A	175.76 (16)	C12B—C13B—C14B—C15B	176.30 (19)
C13A—C14A—C15A—C16A	1.7 (3)	C13B—C14B—C15B—C16B	0.0 (3)
C14A—C15A—C16A—C17A	−2.2 (3)	C14B—C15B—C16B—C17B	1.1 (4)
C15A—C16A—C17A—C18A	1.1 (3)	C15B—C16B—C17B—C18B	−0.8 (3)
C14A—C13A—C18A—C17A	−1.1 (3)	C14B—C13B—C18B—C17B	1.5 (3)
C12A—C13A—C18A—C17A	−176.80 (17)	C12B—C13B—C18B—C17B	−175.93 (18)
C16A—C17A—C18A—C13A	0.6 (3)	C16B—C17B—C18B—C13B	−0.4 (3)

### Hydrogen-bond geometry (Å, °)

**Table:**

Cg3 and Cg6 are the centroids of the of the C13A–C18A and C13B–C18B rings, respectively.

**Table:**

*D*—H···*A*	*D*—H	H···*A*	*D*···*A*	*D*—H···*A*
C12B—H12C···N1A^i^	0.97	2.46	3.422 (2)	172
C5A—H5AA···Cg6^ii^	0.93	2.91	3.842 (2)	178
C12A—H12A···Cg3^iii^	0.97	2.62	3.551 (2)	161

Symmetry codes: (i) −*x*, −*y*+1, −*z*; (ii) −*x*, *y*+1/2, −*z*+1/2; (iii) −*x*+1, −*y*+2, −*z*.
